# Enhanced serodiagnosis of melioidosis by indirect ELISA using the chimeric protein rGroEL-FLAG300 as an antigen

**DOI:** 10.1186/s12879-022-07369-4

**Published:** 2022-04-19

**Authors:** Sumet Wajanarogana, Water R. J. Taylor, Kanyanan Kritsiriwuthinan

**Affiliations:** 1 Department of Basic Medical Science, Faculty of Medicine, Vajira Hospital, Navamindradhiraj University, Bangkok, 10300 Thailand; 2 Mahidol Oxford Tropical Medicine Research Unit, Bangkok, 10400 Thailand; 3 Center for Tropical Medicine and Global Health, University of Oxford, Oxford, UK; 4 Faculty of Medical Technology, Rangsit University, Pathumthani, 12000 Thailand

**Keywords:** Melioidosis, Chimeric protein, Indirect ELISA, rGroEL-FLAG300, *Burkholderia*, Serological diagnosis

## Abstract

**Background:**

The accurate and rapid diagnosis of melioidosis is challenging. Several serological approaches have been developed using recombinant antigens to improve the diagnostic indices of serological tests for melioidosis.

**Methods:**

Fusion proteins from *Burkholderia pseudomallei* (rGroEL-FLAG300) were evaluated as a potential target antigen for melioidosis antibodies. A total of 220 serum samples from 38 culture proven melioidosis patients (gold standard), 126 healthy individuals from endemic (n = 37) and non-endemic (n = 89) Thai provinces and 56 patients with other proven bacterial infections as negative controls were tested using indirect enzyme-linked immunosorbent assays (ELISA).

**Results:**

Using an optical density (OD) cut-off of 0.299148, our assay had 94.74% sensitivity (95% confidence interval (CI) = 82.3–99.4%), 95.05% specificity (95% CI = 90.8–97.7%), and 95% accuracy, which was better than in our previous work (90.48% sensitivity, 87.14% specificity, and 87.63% accuracy).

**Conclusion:**

Our results suggest that the application of chimeric antigens in ELISA could improve the serological diagnosis of melioidosis and should be reconfirmed with greater patient numbers.

## Background

The causative agent of melioidosis, a potentially fatal infectious disease, is the soil-dwelling Gram negative, flagellar bacterium called *Burkholderia pseudomallei*. Melioidosis is endemic predominantly in Southeast Asia and Northern Australia [1, 2]. Sporadic cases have been reported from a range of other countries [3–5] and the distribution of *B. pseudomallei* is predicted in as many as 82 countries. The annual incidence rate of human infection is estimated at 165,000 cases/year with 89,000 deaths [6]. However, owing to the limited availability of laboratory diagnosis, melioidosis is under-recognized. Moreover, its protean clinical manifestations, which range from acute sepsis or pneumonia to a chronic localized infection, challenge early diagnosis and late diagnosis is associated with increased morbidity and mortality [7] even with appropriate antibiotic treatment [8, 9].

Melioidosis is transmitted via skin abrasions, inhalation, or even ingestion [10, 11] but person-to-person transmission can occur rarely [12]. Currently, bacterial culture is the preferred reference diagnostic method but it is slow and has a low sensitivity of ~ 60% [13] and serology detects antibodies against extracted or purified recombinant *B. pseudomallei* protein antigens. The latter have been developed to overcome the limitations of the standard indirect hemagglutination assay (IHA), which has low diagnostic indices in endemic regions (~ 60% sensitivity and specificity) [14–16]. In-house enzyme-linked immunosorbent assays (ELISAs) using recombinant or crude extract antigens such as the outer membrane protein (ompA) [17–19], chaperonin molecule (GroEL) [18], immunodominant flagellin fragment [19, 20], and lipopolysaccharide (LPS) [21, 22], have higher sensitivities (~ 76–90%), and specificities (~ 88–95%) compared to IHA. Most recently, mass spectrometry has been applied for the rapid identification of *B. pseudomallei* [23, 24] but it is costly and its reproducibility needs improvement.

We have shown previously that the variable truncated region of the flagellin protein, FLAG300, of *B. thailandensis* E264 is a useful target for melioidosis antibodies [19, 20] but its expression is low and it is insoluble. Studies on protein expression have shown that when recombinant proteins are co-expressed with chaperones, their yields increase and inclusion body formation is inhibited [25–28]. Moreover, the expression of proteins with large tags as fusion proteins have been widely used to enhance protein production and solubility [29, 30]. The construction of a chimeric protein containing a large chaperonin, GroEL, and FLAG300 is well established for detecting melioidosis antibodies. In order to develop an indirect ELISA, a considerable amount of soluble fusion protein (GroEL-FLAG300) is needed. We, therefore, set out to produce GroEL-FLAG300 and assess its potential as a target antigen to improve the diagnostic indices of melioidosis by indirect ELISA.

## Methods

### Plasmids, bacterial strains, and culture conditions

The *FLAG300* fragment (~ 300 bp) was isolated from our constructed pET17b-FLAG300 plasmid [20], and linked downstream to the pET24a-rGroEL plasmid [18] to generate fusion genes (~ 1900 bp). The recombinant plasmid was first harbored in *E. coli* XL1-Blue for maintenance and then re-transformed into *E. coli* BL21 (DE3) for chimeric protein expression. All bacteria were grown in Luria–Bertani (LB) media (Difco, Detroit, MI, USA) containing 50 μg/mL of kanamycin.

### Serum samples

All human serum samples (n = 220) used in this study were collected and stored anonymized before they were used. The samples were categorized as follows: (1) culture positive melioidosis patient sera (PS) (n = 38), (2) serum from healthy normal volunteers (NS) (n = 126) obtained from endemic (n = 37) and non-endemic (n = 89) provinces of Thailand, and (iii) disease control sera (DS) (n = 56) collected from patients infected with other bacteria from endemic and non-endemic areas: *E. coli* (n = 10), *Klebsiella pneumoniae* (n = 12), *Pseudomonas* spp. (n = 13), gram-positive cocci (n = 7), and other glucose non-fermentative (GNF) bacteria (n = 14).

### Construction of the recombinant plasmid pET24a-rGroEL-FLAG300

Initially, *FLAG300* from our original clone (pET17b-FLAG300) was amplified with a set of primers at *Eco*RI and *Hin*dIII restriction sites for the forward (5′-GAATT CCGGCACGATCAAGGTGGCG-3′) and reverse primers (5′-CCCAAGCTTCTGGTACGC GCCCGT-3′), respectively. Primers were custom-synthesized from Bio Basic Inc., Canada. Next, polymerase chain reaction (PCR) was performed using 50 μL of the final reaction volume containing 50–100 ng of the DNA template, 0.5 μL of 20 mM of dNTPs, 20 pmol of each primer, 1X *Pfu* buffer with MgSO_4_, and 1.25 U of *Pfu* DNA polymerase (Fermentas, Life Sciences, Massachusetts, USA). The PCR mixture was amplified based on the following protocol: preheat at 95 °C for 5 min, followed by 30 cycles of denaturation at 94 °C for 1 min, annealing at 65 °C for 1 min, extension at 72 °C for 1 min, and a final cycle at 72 °C for 5 min. The amplicon and recombinant plasmid (pET24a-rGroEL) were completely double-digested with the *Eco*RI plus *Hin*dIII (Biolabs, Massachusetts, USA) and gel-purified using the GeneJET Gel Extraction Kit (Thermal Fisher Scientific). *Eco*RI-*Hin*dIII DNA fragments were fused with T4 DNA ligase (Biolabs) at 16 °C for 16–18 h. The recombinant plasmid containing fusion genes was designated pET24a-rGroEL-FLAG300 and transferred into competent *E. coli* hosts by heat-shock transformation. The carboxyl end of the chimeric protein (~ 59 kDa of rGroEL plus ~ 13 kDa of FLAG300) was fused with the hexa-histidine tag that was purified by IMAC and identified with anti-his antibody via western blotting.

### Chimeric protein expression (rGroEL-FLAG300), purification, and verification

The transformant carrying pET24a-rGroEL-FLAG300 was cultured in LB-kanamycin medium at 37 °C shaken at 200 rpm for 16–18 h and sub-cultured in 800 mL of freshly prepared LB-kanamycin medium at 1% inoculation rate. With prior induction of protein expression, the bacterial culture was allowed to grow until reaching an optical density at 600 nm (OD_600nm_) of 0.7–0.8. Next, isopropyl-β-d-thiogalactoside (IPTG) (Fermentas, Life Sciences) was added to the final concentration of 1 mM and the medium was further incubated at 16 °C shaken at 100 rpm overnight to produce soluble chimeric protein. The induced culture was then centrifuged at 4700 g for 10 min at 4 °C and the cell pellet formed was collected. The harvested pellet was resuspended in 20 mL of IMAC5 buffer (20 mM Na_2_HPO_4_, 1 M NaCl, 10% [v/v] glycerol, and 5 mM imidazole), including 0.1 mM of phenylmethyl sufonyl fluoride (PMSF) (Bio Basic Inc.) and lysed by sonication in an ice bath. The cell pellet was disrupted 10 times with pulse on at 40% amplitude for 30 s and then pulse off for 30 s. The soluble fraction was separated by centrifugation at 7000 g for 45 min at 4 °C and 2 mL of equilibrated TALON™ resins (Clontech Laboratories Inc., Mountain View, CA, USA) and IMAC5 was added to this fraction to purify the fusion protein by immobilized metal affinity chromatography (IMAC). After incubation for 16–18 h at 4 °C under orbital agitation, the column was set up and continuously washed with 40 mL of IMAC5, 20 mL of IMAC10, 20 mL of IMAC15, and, finally, with 20 mL of IMAC20. All ingredients of IMAC5, IMAC10, IMAC15, and IMAC20 were the same, except for imidazole at 5 mM, 10 mM, 15 mM, and 20 mM, respectively. The bound chimeric proteins were eluted with 10 mL of IMAC400 and concentrated using the Amicon™ Ultra Centrifugal Filter (Millipore Corporation, Burlington, Massachusetts, USA). The eluted protein concentration was determined using the Bradford protein assay by measuring the absorbance at 595 nm (Bio-Rad Laboratories Inc., Hercules, California, USA) and stored at − 80 °C until further use.

To verify the chimeric protein, SDS-PAGE and western blotting were performed. The purified proteins were: (1) separated on 12% polyacrylamide gels by electrophoresis and stained with Coomassie brilliant blue R-250 for SDS-PAGE analysis, and (2ii) transferred onto a polyvinylidene difluoride (PVDF) membrane (Pall Corporation, Port Washington, New York, USA) for western blot hybridization. The blotted membrane was washed twice with TBS buffer (10 mM Tris–HCl; pH 7.5, 150 mM NaCl) and soaked with a blocking buffer (20 mM Tris–HCl; pH 7.5, 500 mM NaCl 0.05% [v/v] Tween 20, 0.2% [v/v] Triton X-100 [TBST] and 5% non-fat dry milk) for 1 h at room temperature. After twice washing with TBST buffer and once with TBS, 1:1000 diluted anti-His horseradish peroxidase [HRP] conjugate (Qiagen, Hilden, Germany), the blocking buffer was added and the mixture was incubated for 1 h. The membrane was washed as in the previous step and visualized for immunoreactivity with 3,3′,5,5′-tetramethylbenzidine (TMB) substrate (KPL, Gaithersburg, MD, USA).

In addition, the chimeric proteins were investigated by western blotting hybridization. The blotted membranes were separately probed with human antibodies from 1:200 dilution of pooled positive sera from the melioidosis patients (n = 5) and pooled normal sera from healthy donors (n = 5). The secondary antibodies from 1:2000 diluted goat anti-human IgG/IgM/IgA HRP conjugate (KPL) were added to react with the immune complex and detected by adding the TMB substrate.

### Determination of the specific binding of chimeric protein with melioidosis antibodies by indirect ELISA

Each serum sample from the three groups (i.e., PS, NS, and DS) was tested in duplicate using the 96-well Microlon™ plate (Greiner bio-one, Kremsmünster, Austria). 100 μL of 10 μg/mL purified protein in the coating buffer (0.05 M carbonate buffer, pH 9.6) was added to the microtiter plate and incubated at 4 °C for 16–18 h. The plate was rinsed with PBST buffer (0.15 M PBS, 0.1% Tween 20, pH 7.4) and incubated with 200 μL of the blocking buffer (2% BSA in 0.15 M PBS, pH 7.4) for 1 h at room temperature to prevent nonspecific binding, followed by washing again with PBST. The immunoreactivity was tested according to the following protocol: 100 μL of 1:3200 diluted sample in the serum diluent (1% BSA in PBST) was added and incubated at 37 °C for 1 h and then rinsed with PBST. 100 μL of 1:10,000 diluted goat anti-human IgG HRP conjugate (KPL) was added to the mixture and then incubated at 37 °C for 1 h. Detecting IgG is better than IgM to diagnose melioidosis by indirect ELISA. The reaction time of 10 min was maintained after adding 100 μL of the TMB substrate and the reaction was later stopped with 50 μL of 2 M H_2_SO_4_. The resultant color product was determined under OD_450nm_ reading with a microplate reader (Bio-Rad model 550, Bio-Rad Laboratories Inc.). For each experimental plate, positive (pooled melioidosis patient sera), negative (pooled normal sera), and direct conjugate controls (diluent without serum) were used.

### Statistical analysis

Data analysis and graphing were performed using the statistical software package SPSS 16.0 for Windows (SPSS) and Stata version 13.0 (StataCorp LP, College Station, Tx). Mean and standard deviation (SD) optical densities (OD) of normal serum samples were calculated. The upper limit of normal was determined as mean plus 2SD and serum samples exceeding this value were considered positive. Using the bacterial culture results as the reference method (“gold standard”) and the NS and DS groups as true negatives, the diagnostic indices (sensitivity, specificity, accuracy) of this developed assay were determined. The independent t-test was used to analyze the difference in the mean OD of each group. A *p* value < 0.05 was considered to be statistically significant.

## Results

### *FLAG300* isolation and recombinant plasmid construction

**Fig. 1:**
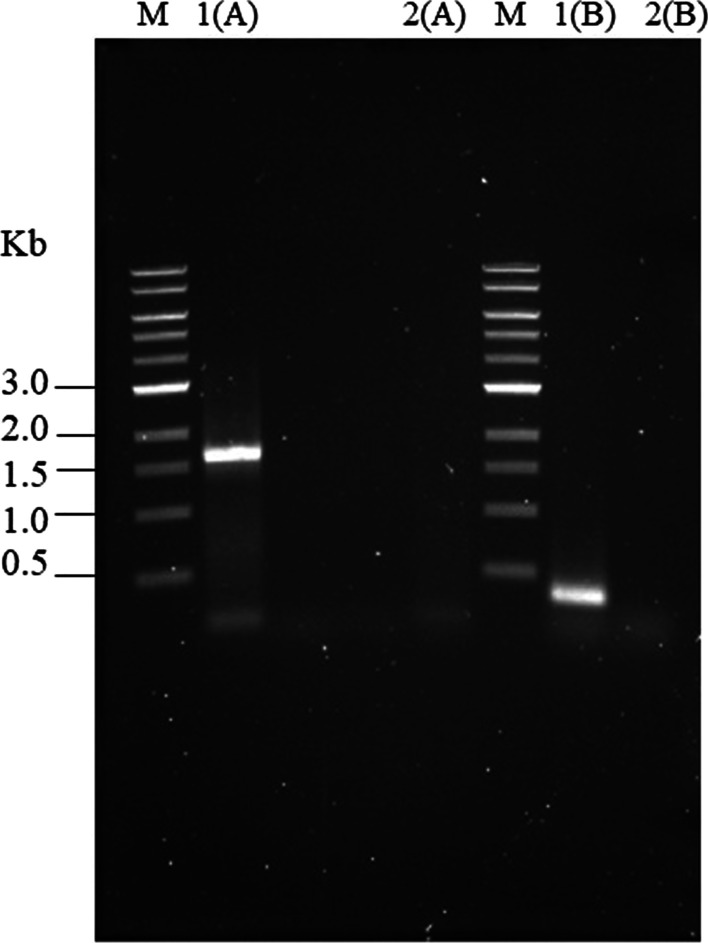
Re-amplification of pET24a-rGroEL-FLAG300 with *GroEL* primers (**A**) and *FLAG300* primers (**B**). M: 1-kb DNA standard marker, 1: positive clone, 2: negative control

An amplified product of approximately 300 bp that covered the variable region of flagellin (*FLAG300*) was attained using the specific primers as described earlier and then absolutely double-digested with *Eco*RI and *Hin*dIII. The purified *FLAG300* fragment was introduced into the pET24a-rGroEL plasmid to construct a designated pET24a-rGroEL-FLAG300 recombinant plasmid. It was then transformed into the expression host *E. coli* BL21 (DE3) and grown on a medium containing kanamycin. The selected clone was confirmed with PCR amplification (Fig. 1). The amplicons were obtained at the exact size (approximately 1600 bp for *GroEL* and 300 bp for *FLAG300*).

### Soluble chimeric protein expression and characterization

**Fig. 2:**
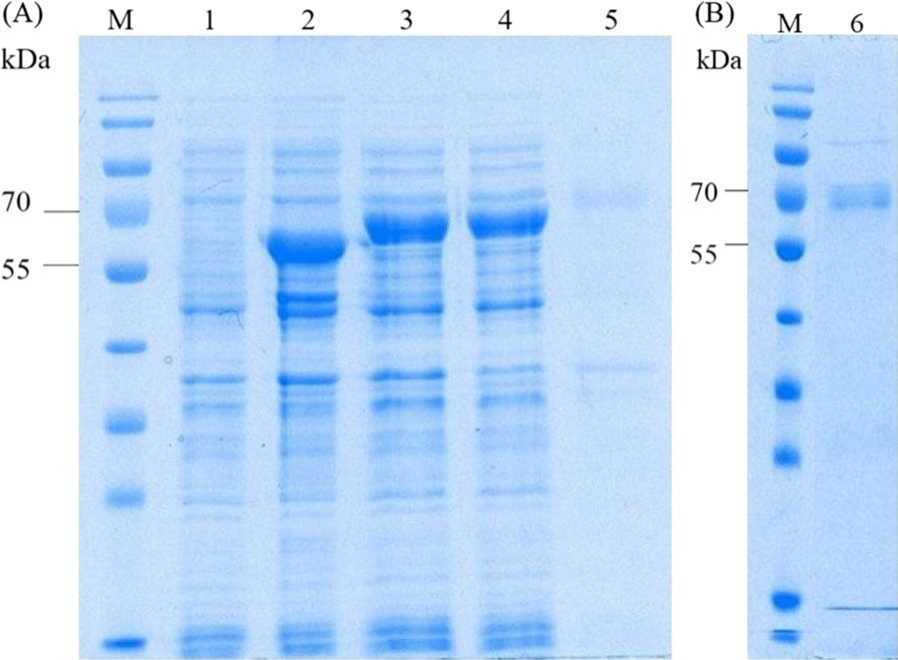
Chimeric protein expression and fractionation (**A**) and purification (**B**). M: protein standard marker, 1, 2, 3: crude protein extracts from non-induced, induced pET24a-rGroEL, and induced pET24a-rGroEL-FLAG300, respectively, 4: soluble fraction, 5: inclusion bodies fraction, and 6: purified protein

**Fig. 3:**
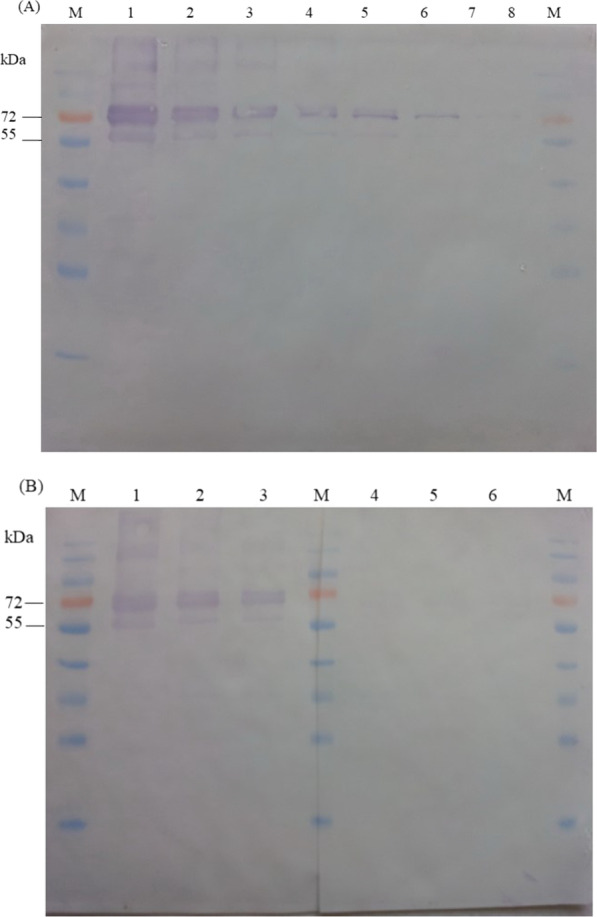
**A** Western blotting to confirm specific immunoreactivity of fusion protein (rGroEL-FLAG300) with an anti-histidine tag antibody. M: pre-stained protein standard marker, 1–8: serial two-fold dilution of purified fusion protein. **B** serial two-fold dilution of recombinant protein probed with pooled positive melioidosis sera (1–3) and with antibodies from pooled normal sera (4–6)

The expression host, *E. coli* BL21(DE3), carrying the construct recombinant plasmid was induced with 1-mM IPTG to express the chimeric protein rGroEL-FLAG300. This expressed protein of was ~ 72-kDa in size and mainly appeared in a soluble fraction (Fig. 2). The chimeric protein was purified by IMAC and identified with anti-his antibody by western blotting (Fig. 3A). Moreover, the immunoreactivity of the soluble chimeric protein was also analyzed by western blotting using probe antibodies from the pooled positive and negative sera (Fig. 3B).

### Evaluation of the rGroEL-FLAG300, fusion protein as an antigen for the serodiagnosis of melioidosis by indirect ELISA

**Fig. 4:**
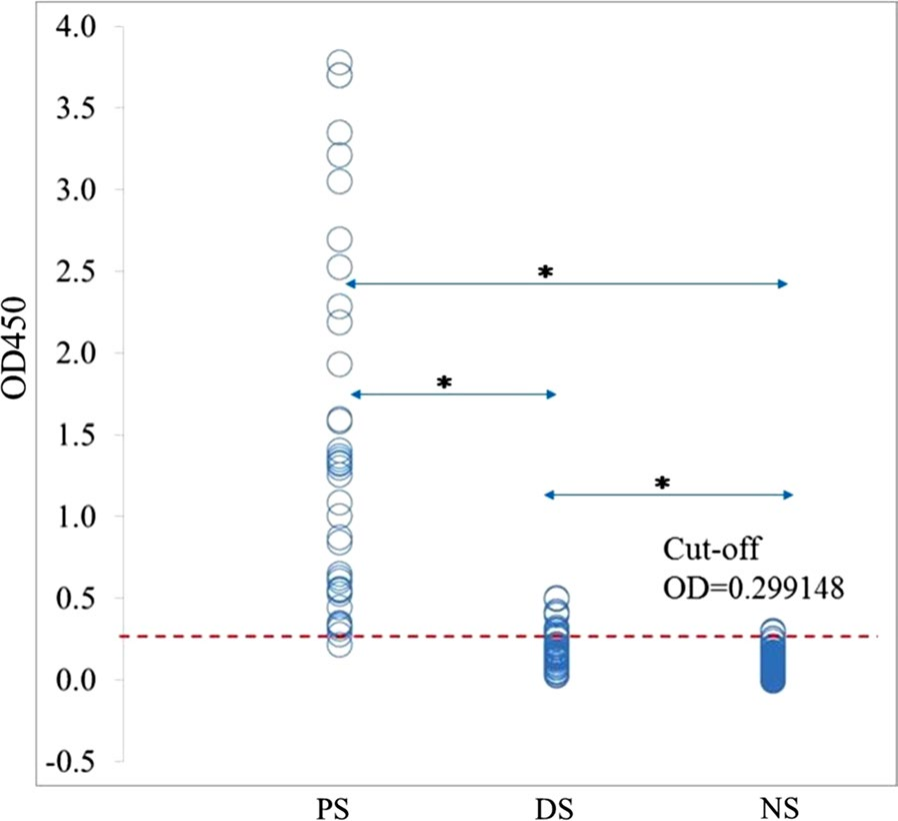
Distribution of the OD values and their cut offs in the three sample groups: patient sera (PS), disease control (DS) and normal sera (NS) groups. *ps < 0.001

The optimized condition of indirect ELISA with the chimeric protein as antigen was performed using all of the 220 human serum samples. The mean and SD of the absorbance values of the NS group were 0.11747 and 0.090839, respectively (Fig. 4). The diagnostic indices of our developed assay were high (> 94%) (Table 1); only 2/38 samples gave false-negative results. There were 9/182 false positive results: one NS group sample (collected in an endemic area) and eight from the DS group: GNF bacteria (5/8), *Pseudomonas* spp. (2/8) and *K. pneumoniae* (1/8). The mean OD values among the 3 sample groups were significantly different (*p* < 0.001, Fig. 4). By contrast, ODs from the endemic and non-endemic areas in the NS group were not significantly different (*p* = 0.504).

**Table 1:** Diagnostic indices of the indirect rGroEL-FLAG300 ELISA

Serum sample group (n)	No. of positive rGroEL-FLAG300 ELISA	Diagnostic indices		
		Sensitivity (%)	Specificity (%)	Accuracy (%)
PS^a^ (38)	36	94.74	95.05	95
DS^b^ (56)	8			
NS^c^ (126) -Endemic area (37)	1			
-Non-endemic area (89)	–			

^a^melioidosis patient sera, ^b^disease control sera, ^c^healthy donor sera

## Discussion

Melioidosis has a high mortality and so the early and reliable diagnosis of melioidosis and appropriate treatment are essential to reduce mortality and morbidity. Various studies have reported improved diagnostic indices with serological tests using recombinant protein antigens over the current clinical standard, IHA. In our previous study, we reported that FLAG300 protein acts as a potential target antigen for melioidosis antibodies. However, the flagellin protein fragment is always expressed as a misfolded insoluble protein in low quantity. To counter these properties, an expression system of fusion proteins with GroEL was applied, which assists with its in vivo folding and improves its yield and bioactivity [31–33].

We successfully produced a soluble chimeric protein, rGroEL-FLAG300, with considerable yield and, before using it to develop the indirect ELISA, we determined its specific binding with melioidosis antibodies by western blotting and noted no cross-immunoreactivity signal in the pooled normal sera. Furthermore, the diagnostic indices of the developed indirect ELISA were evaluated using 3 groups of serum samples and, based on our cut-off, high values of (~ 95%) sensitivity, specificity, and accuracy were obtained. These values are a substantial improvement over those reported by previous studies (90.48% and 92.1% for sensitivity, 87.14% and 88.3% for specificity, and 87.63% and 88.9% for accuracy [18, 19]. Moreover, the mean OD of the PS group was significantly higher than the mean ODs of the NS and DS groups, although this statistical difference was also observed between the DS and NS groups. The sera of patients from the DS group showed cross-reactivity to chimeric proteins of rGroEL-FLAG300, suggesting Gram negative bacteria may share certain immuno-dominant epitopes as melioidosis and so limit the usefulness of rGroEL-FLAG300 as a diagnostic protein. Further validation of rGroEL-FLAG300 should be carried out with sera from other diseases that share similar clinical features to melioidosis such as leptospirosis, tuberculosis, dengue, chikungunya, and rheumatoid arthritis. Various attempts have been made using individual recombinant proteins to develop a serological test for melioidosis resulting in quite high diagnostic indices (88–95% of sensitivity and 93–98% of specificity) [17, 34–36]. We suggest that a fusion recombinant protein could improve the serological diagnosis of melioidosis and its utility should also be considered in the design of a vaccine against *B. pseudomallei* infection. The flagellin fragment is a potential antigen and GroEL acts as an adjuvant to stimulate the innate and adaptive immune response [37–40]. Experience with vaccines used for animal immunization and anti-tumor immunity show that immune responses are significantly increased by using fusion antigens [41, 42]. Therefore, the next-generation anti-melioidosis vaccine should adopt genetic fusion to produce a chimeric protein as an antigen adjuvant.

## Conclusions

Data from our small study showed promising results for the indirect ELISA using the chimeric protein rGroEL-FLAG300. More work is needed to confirm our findings and ascertain the extent of false positive results due to cross reaction. Moreover, rGroEL-FLAG300 can be expressed in a soluble form in large quantities for use as an antigen to develop other diagnostic platforms.

## Data Availability

All data generated or analysed during this study are included in this published article.

## Abbreviations

BSA: Bovine serum albumin
bp: Basepair
DS: Disease control sera
ELISA: Enzyme-linked immunosorbent assay
FLAG300: Variable region of flagellin
GNF: Glucose non-fermentative
HRP: Horseradish peroxidase
IHA: Indirect hemagglutination assay
IMAC: Immobilized metal affinity chromatography
IPTG: Isopropyl-β-d-thiogalactoside
kDa: Kilodalton
LB: Luria–Bertani
LPS: Lipopolysaccharide
NS: Healthy donor sera
OD: Optical density
Omp: Outer membrane protein
PBS: Phosphate buffer saline
PBST: Phosphate buffer saline tween
PCR: Polymerase chain reaction
PMSF: Phenylmethyl sufonyl fluoride
PS: Patient sera
PVDF: Polyvinylidene difluoride
rGroEL: Recombinant GroEL
SD: Standard deviation
SDS-PAGE: Sodium dodecyl sulfate polyacrylamide gel electrophoresis
TMB: 3,3′,5,5′-Tetramethylbenzidine

## Declarations

### Completing interests

The authors declare that they have no completing interests.
